# The stuff that dreams are made of: HIV‐positive adolescents’ aspirations for development

**DOI:** 10.1002/jia2.25057

**Published:** 2018-02-27

**Authors:** Rebecca Hodes, Jenny Doubt, Elona Toska, Beth Vale, Nompumelelo Zungu, Lucie Cluver

**Affiliations:** ^1^ AIDS and Society Research Unit University of Cape Town Cape Town South Africa; ^2^ Department of Social Policy and Intervention University of Oxford Oxford United Kingdom; ^3^ Mapungubwe Institute for Strategic Reflection Johannesburg South Africa; ^4^ Human Sciences Research Council Pretoria South Africa

**Keywords:** HIV‐positive adolescents, participatory research, Sustainable Development Goals, development interlinkages, health access, food security, water and sanitation

## Abstract

**Background:**

The Sustainable Development Goals (SDGs) commit to strengthening collaborations between governments and civil society. Adolescents are among the key target populations for global development initiatives, but research studies and programmes rarely include their direct perspectives on how to promote health and wellbeing. This article explores how both the methods and the findings of participatory research provide insights into adolescents’ aspirations across the domains of health and social development. It investigates how adolescents conceive of health and social services as interconnected, and how this reflects the multisectoral objectives of the SDGs.

**Methods:**

This research was conducted within a longitudinal, mixed‐methods study of HIV‐positive adolescents (n = 80 qualitative participants, n = 1060 quantitative interviews). Between November 2013 and February 2014, a participatory exercise – the “dream clinic” – was piloted with 25 adolescents in South Africa's Eastern Cape. Key themes were identified based on the insights shared by participants, and through visual and thematic analysis. These findings were explored through a second participatory exercise, “Yummy or crummy? You are the Mzantsi Wakho masterchef !,” conducted in January 2016. Findings are described in relation to emerging quantitative results.

**Results:**

Mixed methods explored associations between access to food, medicines, clean water and sanitation in HIV‐positive adolescents’ aspirations for development. The exercises produced practicable recommendations for innovations in development, based on associations between healthcare, food security, clean water and sanitation, while illustrating the value of partnership and collaboration (the objective of SDG17). Findings capture strong interlinkages between SDGs 2, 3 and 6 – confirming the importance of specific SDGs for HIV‐positive adolescents. Study results informed the objectives of South Africa's National and Adolescent and Youth Health Policy (2017).

**Conclusions:**

Participatory research may be used to leverage the perspectives and experiences of adolescents. The methods described here provide potential for co‐design and implementation of developmental initiatives to fulfil the ambitious mandate of the SDGs. They may also create new opportunities to strengthen the engagement of adolescents in policy and programming.

## 1 Introduction

### 1.1 Public participation and the SDGs

The Sustainable Development Goals (SDGs) are designed to promote economic, social, and environmental wellbeing on a global scale 1, 2. In designing the SDGs, the United Nations sought to elicit the perspectives of vulnerable and marginalized groups, including diverse members of civil society, alongside consultations with government partners 3, 4. The importance of inclusivity was noted in the process of both conceiving 5 and prospective monitoring of the goals 6, and is further articulated in SDG 16, which includes a commitment to inclusivity in the promotion of peace, justice, and the building of strong and responsive institutions. But how is this greater inclusivity to be practicably realized, particularly among “hard‐to‐reach populations” such as adolescents?

The necessity of improving the effectiveness and scalability of health programmes for adolescents is apparent in numerous health and development indicators, including those that focus on HIV transmission and treatment. A growing research base demonstrates high rates of defaulting from antiretroviral treatment (ART) among adolescents in public settings 7, 8. The effectiveness of HIV treatment does not depend solely on the provision of medicines and healthcare, but is also linked to food security and access to health facilities 8, 9, 10. The successes of health and social initiatives for adolescents depend further on their ability to respond to adolescent needs in ways that resonate with their own ideas about, and aspirations for, health and wellbeing.

This article describes how both the methods and the findings of participatory exercises may provide insights into adolescents’ aspirations for development. Combining data from the qualitative and quantitative components of the Mzantsi Wakho study, it explores how adolescents conceive of health and social services as interconnected. It questions how the domains of health and social development intersect for adolescents, and what this reflects about the multisectoral objectives of the SDGs.

### 1.2 Adolescents and the SDGs

Creative forms of participatory research may be used to expand the evidence base within public health and development to include those who are marginalized by socio‐economic and structural circumstances, such as poverty, gender and disability 11. These methods may foster greater reflexivity between researchers and subjects 12, helping to bridge divides between global and national policy‐makers and programmers, and local beneficiaries. Participatory research methods include visual or performance‐based data collection tools that enable participants to document and analyse their experiences, identify solutions to local problems, and critically assess development initiatives 13, 14, 15, 16.

## 2 Methods: Participatory Research and The Mzantsi Wakho Study

This article focuses principally on a participatory research exercise entitled the “dream clinic”, triangulating findings with a second participatory exercise, “Yummy or crummy? You are the Mzantsi Wakho masterchef!.” We combine results from these exercises with wider themes and emerging findings from a mixed methods, cohort study about youth health in South Africa. The study name, “Mzantsi Wakho” – meaning “Your South Africa,” captures its intention to engage youth in conceiving and relating their own goals for health and social development.

Mzantsi Wakho is a partnership of qualitative and quantitative researchers. The study is advised by the South African Departments of Basic Education, Health, Social Development and the Human Sciences Research Council, bilateral agencies UNICEF and UNAIDS, and non‐governmental organisations, including Pediatric‐Adolescent Treatment for Africa (PATA). These partnerships have informed the study's focus on interconnections between the domains of health and social development for adolescents.

Starting in 2013, the study has combined multiple qualitative methods, including in‐depth interviews, observations and focus groups, to investigate the healthcare practices and experiences of adolescents and young people 17, 18, 19, 20. From 2014 to 2015, the study established a quantitative cohort of 1060 HIV‐positive 10 to 19 year‐olds. A structured questionnaire captures the health and social factors associated with medicines‐taking and sexual health 8, 9. The sample was 55% female, and had a mean age of 13.8. 97% of participants spoke isiXhosa as their first language. About 19% lived in informal housing, and 21% were based in rural areas. Nearly half were maternal orphans (44%) and 30% paternal orphans. All HIV‐positive participants had been initiated onto ART, with an average of 5.9 years on treatment. 75% knew their HIV‐positive status 21, defined as having been disclosed to by an adult caregiver or healthcare worker, and by adolescent self‐reported knowledge of HIV‐positive status and understanding ART as medicine to treat HIV 22, 23. Findings from both the qualitative and quantitative components of the study informed the adaptation and integration of research tools with multiple sources of data analysed by inter‐disciplinary investigators 22, 23, 24, 25.

Due to the legal and ethical challenges of working with young people, studies about health often use adults as “proxies” for adolescent experiences. Mzantsi Wakho's approach is different: positioning adolescents as the primary experts on their own health behaviours, conducting research both within and beyond clinical contexts, in homes and in leisure spaces, and seeking new ways of documenting adolescents’ experiences and perspectives. Ethical approval for this study was provided by Research Ethics Committees at the Universities of Oxford (SSD/CUREC2/12‐21) and Cape Town (CSSR 2013/4), Eastern Cape Departments of Health and Basic Education, and ethical review boards of participating hospitals. The study follows a deliberative approach to ethical permissions, seeking ongoing guidance to ensure consent and protect confidentiality during primary research, analysis, and dissemination.

### 2.1 “Dream clinics”

The “dream clinic” used visual media to capture and convey adolescents’ aspirations for health and social services. The exercise drew on the utility of participatory, socio‐spatial mapping exercises as research tools 14, 26. The exercise was piloted in a workshop held in the Eastern Cape, in November 2013, with 9 adolescents from a rural area. It was repeated with 16 adolescents from a peri‐urban area within the same health district in February 2014. Adolescent participants of mixed gender, ranging in age from 10 to 19, were recruited from local community‐based organizations that provided HIV care and treatment. Adolescents in the first workshop knew their HIV‐status, were openly disclosed, and knew one another's status as a consequence of being in the same support group. The second workshop combined openly‐disclosed, partially‐disclosed and undisclosed adolescents, and no specific references were made to HIV or to ART. For adolescents younger than 18‐years, voluntary informed consent for participation was obtained from caregivers, alongside voluntary, informed assent from adolescents.

The “dream clinic” exercise used a series of open‐ended prompts to facilitate adolescents in designing and drawing their ideal health facilities. Adolescents were invited to imagine the location and structure of the clinic, and to recreate its surroundings and interior. The exercise was conducted in three languages – isiXhosa, English, and Afrikaans. Facilitators gave prompts principally in English and isiXhosa, with additional explanations given to individuals and groups in their primary languages. All facilitators were trained on how to engage adolescent participants, including how to avoid dominating or directing participation. Participants chose to work alone, or within groups of two to five. Groups included a dispersion of participants according to age and gender, and produced a total of fourteen “dream clinic” illustrations (10 individual drawings in the first workshop, and four group drawings in the second).

At the end of the exercise, each drawing was presented to the broader group, with participants explaining its particular features and significance. Researchers made notes of participants’ responses and interpolations. One of the challenges of this exercise was that many adolescents began by drawing their clinics as they existed. Distinguishing reality from aspiration in analysing the drawings could therefore be difficult. Thematic notes helped to convey participants’ intentions and to differentiate between what they hoped for, and what they experienced directly. Following Martin‐Hilber *et al*. 27, notes were later collated and compared, and key themes identified based on the insights shared by participants, and through visual and discursive analyses of the drawings. Themes identified through the “dream clinics” were explored further with participants through participatory research on the experiential components of medicines‐taking, including through the “Yummy or crummy” exercise described below.

### 2.2 Yummy or crummy?

From November 2015 to January 2016, we designed a participatory research tool to explore the experiential components of medicines‐taking. Named “Yummy or crummy? You are the Mzantsi Wakho masterchef !,” the exercise combined role‐playing with the preference‐ranking features of social media forums. Drawing on the rubric for participatory research developed by Skovdal and Cornish 13, it merged “linkages and relationship tools,” “experiential tools,” and “prioritization and quantification tools.” Through incorporating visual and performative components, the exercise aimed to provide participants with new ways to relate the multisensory experiences of medicines‐taking. Feedback forms used various techniques for assessing medicines‐preferences among young patients 28, including emoticons from social media applications. The content of forms was transcribed, translated, and coded, with key themes identified collectively by researchers who designed and facilitated the exercise. It was piloted with a group of adolescents and young adults (n = 17, male 7, female 9), part of the Teen Advisory Group (TAG), in January 2016. TAG was established within the Young Carers study in 2008, and participants played an advisory role in the Mzantsi Wakho study, taking part in annual workshops from 2012. “Yummy or crummy?” findings are used here to triangulate “dream clinic” findings, with a focus on the intersection of health programming with sanitation and social development from the perspectives of HIV‐positive adolescents.

## 3 Results

### 3.1 Food and water

**Figure 1:**
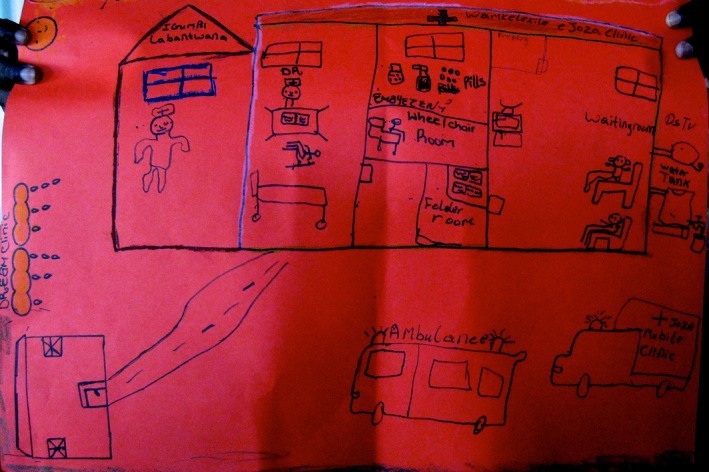
A “dream clinic” (November 2013), including water tanks and taps (far right), a wheelchair room (centre) and an ambulance and mobile clinic (bottom right).

**Figure 2:**
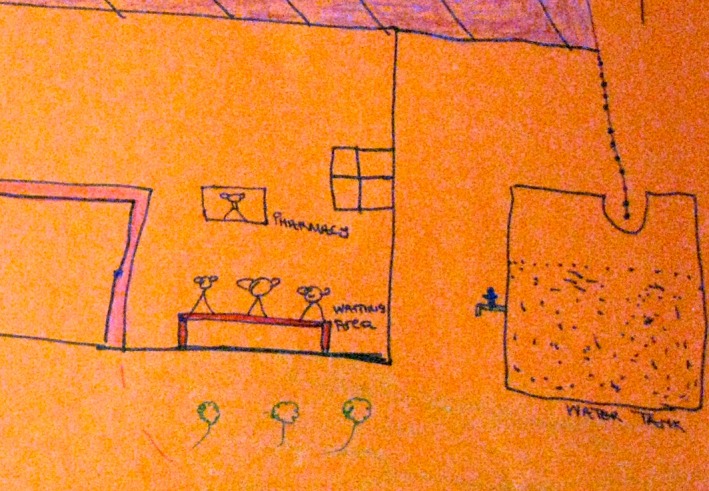
Detail from a “dream clinic” (November 2013) shows a large and full water tank and a tap adjacent to the patient's waiting area.

**Figure 3:**
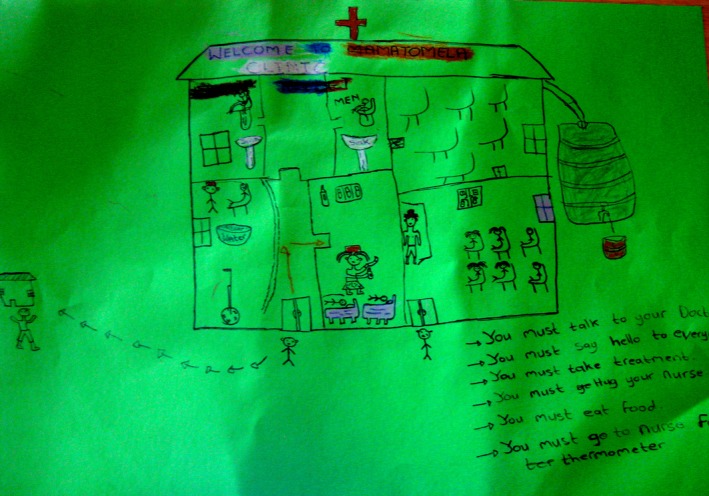
A “dream clinic” (November 2013) features a water tank and a bucket for easy distribution (far right), separate and well‐appointed ablutions for men and women (with taps and toilets, centre top), and a basin with water in the consulting room (centre).

**Figure 4:**
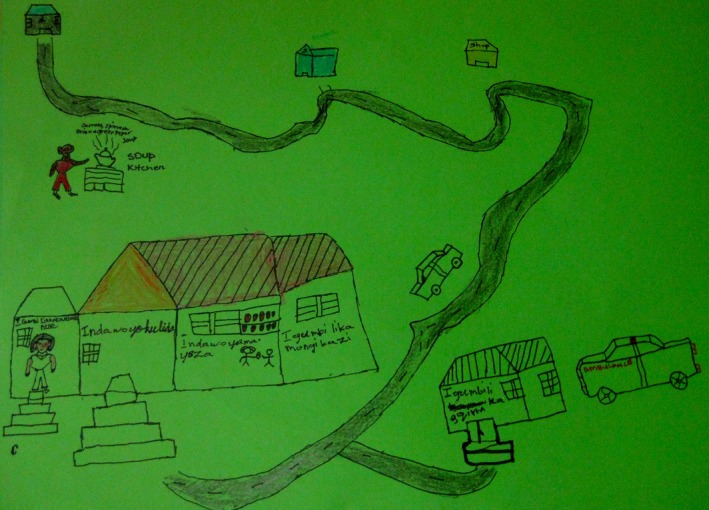
Detail from a “dream clinic” (February 2014) shows the route to the clinic from participants’ homes. The first stop on the route is a soup kitchen, the second is a school.

In numerous “dream clinics”, participants drew food gardens growing next to facilities, with ready access to fresh produce. Bathrooms with sinks and taps, and brimming water tanks, were portrayed (Figures 1, 2, 3). Most drawings included a “tuck shop” or kiosk and food garden in close proximity to the clinic. In one drawing, the entrance to the clinic was pictured not at the clinic's centre, but at its side, next to a soup kitchen (Figure 4). Adolescents’ desires for comprehensive healthcare were conceived in relation to adequate nutrition and food security.

Both within one‐on‐one discussions between facilitators and individual participants, and within group discussions conducted as part of the “dream clinic” exercise, the provision of food and clean water was prioritized (group discussion, “dream clinic” exercise, Mzantsi Wakho workshops November 2013; February 2014). Within the drawings themselves, soup kitchens, tuck shops, and gardens were rendered with the same intricacy and precision as pharmacies and folder rooms within clinics. The inclusion of food and water supplies as features of the “dream clinic” illuminates both the value and the potential scarcity of these resources.

Findings from other components of the Mzantsi Wakho study emphasized the importance of food and water as pre‐requisites for adherence to medicines, suggesting an inter‐linkage between SDGs 2 (food security), 3 (health), and 6 (water and sanitation). Over a fifth (22.6%) of the 1060 HIV‐positive adolescents in the sample reported hunger and food insecurity 9. Many participants relied on school feeding programmes as vital nutritional support, with 93% reporting receiving at least one meal a day at school.

The “Yummy or crummy?” exercise reified adolescents’ emphasis on access to water and sanitation, not just as a medium for swallowing pills, but of agency and mobility. In areas that lacked running water, adolescents on ART were required to plan their movements to ensure that they were close to a water supply at dosing times – especially as their regimens commonly included numerous large pills, making “dry swallowing” difficult. This curtailed the mobility of adolescents in areas with restricted access to water and sanitation where they were, in essence, tethered to taps.

### 3.2 Access

All of the dream clinic drawings portrayed transport routes as roads which were smoothly paved and well‐marked. A short distance between healthcare facilities and homes conveyed some participants’ desire for proximate, readily accessible facilities, although in‐depth interviews conveyed the desire of other participants for facilities distanced from their homes, to ensure greater privacy and avert potential HIV‐related stigma. Wheelchairs were represented in one drawing, relaying the need for equipment and healthcare services for those with disabilities and injuries that impaired movement. Ambulances, pictures in numerous drawings, conveyed the desire for mobile services. Some participants felt, however, that mobile clinics would be less reliable than “fixed” facilities, and the misuse of ambulances as private taxis was reported.

The “dream clinics” revealed adolescents’ aspirations for health services as interconnected with education and social services. One “dream clinic” featured a long and winding road. The first stop on the route to the clinic was a soup kitchen (Figure 4). The second stop was a school. Within this rendition, access to food, education, and healthcare were associated.

### 3.3 Comprehensive services

Representations of clinics as sources of food, water, and social services reflected adolescents’ aspirations for a strong, supportive, and integrated health service that could meet the diverse needs of its users. The clinic was imagined as a site of multiple, interlinking forms of care and support. Reflecting during a workshop (November 2013) on what clinic staff should be expected to do, one adolescent participant gave the following list: “lend us money to go home, give us some lunch… give us pills, take blood tests.” Another participant stated: “I would like the clinic to have food, water; to have medication because they don't give medication; clean toilets; gardens for veg !”

**Figure 5:**
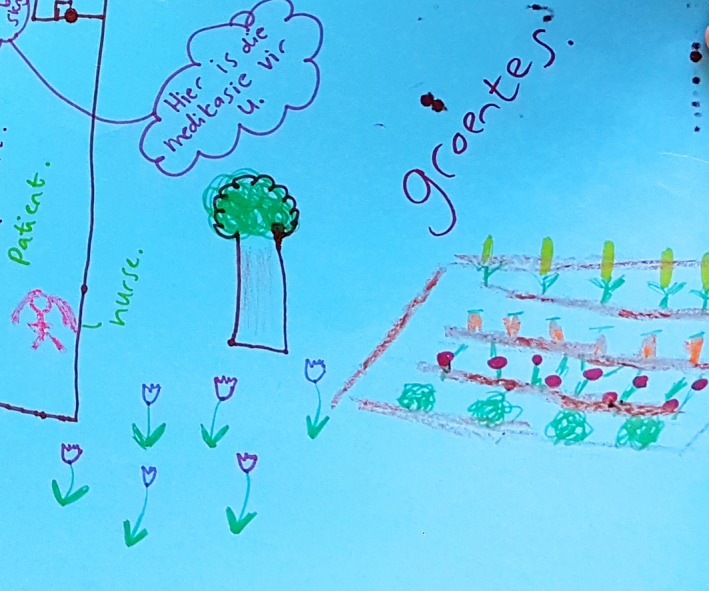
Detail from a “dream clinic” (February 2013) shows a flourishing vegetable garden and plants and trees next to the healthcare facility. It portrays a respectful dialogue between a nurse and patient.

Desires for food security and access to clean water were represented concurrently with objectives for accessible and comprehensive health services, such as well‐stocked pharmacies and ample staff. In Figure 5, a group of participants envisioned a flourishing vegetable garden (“groentes” in Afrikaans) next to an image of nurses dispensing medicines to patients. These explicit linkages between food, water, and pill‐taking reflect a related finding in the Mzantsi Wakho study: that adolescents perceived ability to swallow pills and thus adhere to their ART regimens as reliant on having clean water and “good” food to eat.

### 3.4 Participation

In Figure 5, the speech bubble (in Afrikaans) captures a conversation between a nurse and a patient. Using the formal and respectful tense pronoun, “u,” as opposed to the casual “jy,” the implication is that patients wish to be addressed respectfully by healthcare workers. Adolescents aspired for greater agency in their interactions with healthcare workers, which resonated with the commitment of SDG 16 to create more effective, accountable and inclusive institutions and partnerships for development. While focused on their own priorities in the clinic setting, adolescents in this exercise also (sometimes reluctantly) paid attention to the needs of other vulnerable groups, particularly young mothers and patients with disabilities.

## 4 Discussion

Participatory visual methods have numerous limitations, including questions regarding interpretation, validity, reliability, and integration with other datasets and disciplinary approaches. However, in their potential to engage adolescents directly in the research sphere, participatory techniques have much to offer. This includes the means of exploring experiential associations and aspirations, and of translating research beyond surveys and researcher‐led interviews or focus groups, to forms of investigation that offer participants new channels of engagement and representation. As such these forms of enquiry may provide a means to pursue the more “human‐rights based approach” 29 called for in implementing and evaluating the SDGs. They offer another opportunity to include the views and perspectives of marginalized population groups such as HIV‐affected adolescents, and to design responsive and effective programmes accordingly.

Findings from the “dream clinics” and “Yummy or crummy?” reveal synergies between healthcare provision and access, infrastructure, water and sanitation, and nutrition. Food and water are critical to adherence for participants in this study, interlinking SDGs 2 (food security), 3 (health), and 6 (water and sanitation). This finding is similar to another study conducted in South Africa, in which HIV‐positive patients hoped to access food and drink, such as bread and tea, while waiting in clinic queues. This was not just to staunch hunger built up in the course of long delays to see healthcare workers, but also to eat something before taking ART 30. Associations between hunger, thirst and ART defaulting are documented in the growing literature on ART adherence 31. Within the Mzantsi Waho study, adolescents who reported food insecurity were nearly twice as likely to report past‐week non‐adherence 8. It is increasingly evident that a lack of food is associated with perceived side‐effects from ART, and with missing doses 32, 33, 34.

These findings should be considered in the context of the SDG agenda given the requirement to address the “interconnected factors” of the SDGs, and to explore the experiences of marginalized constituencies, including children and adolescents 5. Understanding what the intended recipients of development initiatives want and need, and partnering with them in their design, adaptation and implementation, is imperative to realize the ambitious objectives of the SDGs.

The participatory exercises described here encouraged a greater plurality of conceptions of healthcare services and their potential improvement 35. Findings from the “dream clinic,” translated into policy recommendations, were incorporated in South Africa's Adolescent and Youth Health Policy for 2017 36, and the UNICEF‐led platform, All‐In to #EndAdolescentAIDS 37. The form and the findings of these “dream clinics” have therefore influenced policy and programming at national and regional levels, propelling the experiences and aspirations of adolescents directly into policy goals and programmatic recommendations. This exercise has been repeated in skills‐development programmes for adolescents and healthcare workers in South Africa by other organizations.

## 5 Conclusion

The formulation of the SDGs reflects a participatory impetus of the broader development agenda. This study proposes ways to include of adolescents in implementing and monitoring the SDGs.

In addition to more structured and traditional research methods, Mzantsi Wakho's “dream clinic” and “Yummy or crummy?” exercises aimed to combine the components of participatory research in partnership with HIV‐positive teenagers in South Africa, encouraging a greater plurality of conceptions of healthcare interventions and their potential improvement. Methods such as these provide an opportunity for researchers, programme implementers, and government representatives to bridge the gap between the rhetorical commitment to broad‐based and inclusive partnerships for development, and their practicable rendition.

Findings from the exercises captured how participants conceived of healthcare as far broader than access to medicines or clinical care. Rather, participants imagined healthcare as part of a developmental lattice that connects sound infrastructure, access to education, and nutrition. Adolescents conceived of themselves as partners in the design and implementation of development initiatives – and as a binding force within this lattice.

## Competing interests

The authors declare that they have no competing interests.

## Authors’ contributions

RH, JD, ET, BV and LC conducted primary research and analysis. NZ guided methods and analysis. All authors wrote, edited and approved the final manuscript.

## Funding

This study was supported by the Nuffield Foundation under grant CPF/41513, the International AIDS Society through the CIPHER grant (155‐Hod), Janssen Pharmaceutica N.V., part of the Janssen Pharmaceutical Companies of Johnson & Johnson, Evidence for HIV Prevention in Southern Africa (EHPSA), a UK aid programme managed by Mott MacDonald (MM/EHPSA/UCT/05150014), the Economic and Social Research Council (IAA‐MT13‐ 003), the Regional Inter‐Agency Task Team (RIATT‐ESA) for Children Affected by AIDS in Eastern and Southern Africa, UNICEF, UNFPA, the European Research Council under the European Union's Seventh Framework Programme (FP7/2007‐2013)/ERC grant agreement no. 313421, the Oak Foundation (R46194/AA001), the Claude Leon Foundation, the John Fell Fund (103/757) and the Philip Leverhulme Trust (PLP‐2014‐095).
